# Allergic rhinitis, rhinoconjunctivitis and hayfever symptoms among children are associated with frequency of truck traffic near residences: a cross sectional study

**DOI:** 10.1186/s12940-015-0072-1

**Published:** 2015-10-26

**Authors:** Joyce Shirinde, Janine Wichmann, Kuku Voyi

**Affiliations:** Department of Environmental Health, Tshwane University of Technology, Private Bag X680, Pretoria, 0001 South Africa; School of Health Systems and Public Health, Health Sciences Faculty, University of Pretoria, P.O. Box 667, Pretoria, 0001 South Africa

**Keywords:** Allergic rhinitis, Rhinoconjunctivitis, Hayfever, Traffic, Air pollution, South Africa

## Abstract

**Background:**

Allergic rhinitis (AR) is an increasing and common condition affecting many people globally, especially children. The aim of the study was to investigate the association between the frequency of truck traffic and allergic rhinitis symptoms, rhinoconjunctivitis and hayfever among 13 to 14 year old school children in Ekurhuleni Metropolitan Municipality, Gauteng Province, South Africa.

**Methods:**

In a cross-sectional study design, 3764 children from 16 randomly selected high schools were eligible to participate, 3468 completed the International Study of Asthma and Allergies in Childhood (ISAAC) Phase I questionnaire of which 3424 were suitable for analysis; the overall response rate was 92 %. Data were analysed using multilevel logistic regression analysis.

**Results:**

The prevalence of self-reported rhinitis ever, current rhinitis rhinoconjunctivitis and hayfever was 52, 40, 21 and 37 % respectively. Rhinitis ever, current rhinitis and current rhinoconjunctivitis were significantly associated with the frequency of trucks passing near residences almost all day on weekdays, (OR 1.46 95 % CI: 1.16 − 1.84), (OR 1.60 95 % CI: 1.24–2.02) and (OR 1.42 95 % CI: 1.09–1.84) respectively. No association was observed between truck traffic and hay fever in the multiple analyses.

**Conclusion:**

The study shows a high prevalence of allergic rhinitis symptoms amongst children. The results support the hypothesis that traffic related pollution plays a role in the prevalence of allergic rhinitis symptoms in children residing in the area.

## Background

Allergic rhinitis (AR) is a global health problem, affecting many people from childhood to adulthood [1]. The disease is most common and one of the leading chronic conditions in children less than 18 years of age; it is frequently ignored, under-diagnosed, misdiagnosed or mistreated [2, 3]. Rhinitis is defined as the inflammation of the nasal lining, but is characterised by nasal symptoms of: sneezing, itching, rhinorrhoea/nasal running and/or nasal congestion [4]. Rhinitis is frequently accompanied by symptoms involving the eyes, ears and throat, including postnasal drainage [2, 4]. The disease affects 400 million people worldwide, with high prevalence recorded in industrialised nations. Epidemiological surveys have reported an increase in the disease, with different regions of the world reporting prevalence rates of between 10 and 40 %. The prevalence of childhood AR shows wide global variation, ranging from 0.8 to 39.7 % [5–10].

The reasons for the global increase in the prevalence of allergic rhinitis are still not understood. The disease has been associated with various risk factors including among others: gender, housing characteristics, socioeconomic status, environmental air pollution, exposure to tobacco smoke, birth during pollen season, no older siblings, exposure to allergens such as animal dander and dust mites [2, 3, 11, 12]. The findings are inconsistent and the major determinant contributing to the development of allergic rhinitis is still unclear. Few air pollution studies have addressed allergic rhinitis as an endpoint with some suggesting that exposure to air pollutants may increase its risk [13]. Studies have also reported that living in closed proximity to a major road with high volumes of motor vehicles or truck traffic is associated symptoms of allergic diseases, due to high levels of air pollutants from traffic [14]. The majority of epidemiological studies emanate from developed countries; little is known about such association in developing countries. In South Africa, AR is an important and common condition encountered in most communities and affecting anywhere from 20 to 30 % of the population. However, data from South Africa is limited, with infrequent updates on circulating aeroallergens and the possible impact of climate change [15].

Existing studies are not generalised; some have small sample sizes and assess specific populations [16, 17]. One study that was carried out in Cape Town, Western Cape Province, reported an increase in the symptoms over a 7 year period, from 30.4 % in 1995 to 38.5 % in 2003 [18]. The main aim of this study was to investigate the association between traffic related-air pollution and allergic rhinitis, current rhinoconjunctivitis and hayfever symptoms amongst children attending schools in Tembisa and Kempton Park areas of Ekurhuleni Metropolitan Municipality (EMM), Gauteng Province, South Africa.

## Methods

### Study area

The study was conducted in Tembisa and Kempton Park areas, which fall under the EMM. Tembisa is the second largest township in Gauteng Province, with both formal and informal housing, being home mainly to people belonging to Black/African ethnic groups. The main air polluting sources in the area includes, amongst others, residential fuel burning (particularly coal), industrial and commercial fuel burning (coal-fired boilers in close proximity to residential areas) and vehicular exhaust emissions (both petrol and diesel) [19]. Kempton Park is a suburban area surrounded by industry and arterial roads connecting Gauteng Province. The OR Tambo International Airport, which is Africa’s busiest airport, is also located nearby. Vehicular exhaust emissions (both petrol and diesel), industrial and commercial fuel burning (coal-fired boilers in close proximity to residential areas), OR Tambo International Airport (contributing a small fraction of low level, concentrated NO_2_) and large industries associated with various stack, vent and fugitive emissions were identified as significantly contributing to air pollution [19]. The (EMM) where the two areas are located falls under the Highveld Region, which was declared an air pollution priority area in the country, due to poor air quality, which is still the worst to date [20].

### Study design, population and sample selection

A cross-sectional epidemiological study was conducted between February and June 2012, following the International Study of Asthma and Allergies in Childhood (ISAAC) Phase I protocol [21]. The ISAAC was designed as a multicentre-study to investigate the epidemiology of asthma, rhinitis and atopic dermatitis amongst children using standardised definitions, allowing comparisons worldwide [21]. A list of all schools (primary and secondary) in EMM was provided by the Gauteng Department of Education. All primary schools were excluded and 16 high schools were randomly selected from the list of high schools. Each school was contacted and requested to participate in the study. Following approval by the principal and governing body in each school, all eligible children between the ages of 13 and 14 years and in Grade 8 were requested to participate. The 13 to 14 year age group was chosen because it is the age most adolescents go to school regularly, making data collection easier. Each school was requested to make available a copy of class lists. An appointment was scheduled with the school to deliver the consent forms for the children two weeks prior to the study and they were requested to return them within three days. The study population consisted of 3764, children based on the numbers given by each school prior to data collection. Data were collected using the English version of ISAAC written questionnaires. The questionnaires were completed by the children in the classroom under the supervision of the data collectors, who were specifically trained and briefed to avoid explanations which could interfere with the participant’s answers.

### Health outcomes

In this study we estimated health outcomes on the basis of positive answers from the written ISAAC questionnaire for 13 to 14 years old. Answers to written questions were self-reported by children. Questions on symptoms relating to rhinitis were as follows:

1. Rhinitis ever: Have you ever had a problem with sneezing or runny or blocked nose, when you DID NOT have a cold or flu? (Yes/No)
2. Current rhinitis: In the past 12 months, have you had a problem with sneezing or a runny or blocked nose, when you DID NOT have a cold or the flu? (Yes/No)
3. Current rhinoconjunctivitis: In the past 12 months, has this nose problem been accompanied by itchy-watery eyes? (Yes/No)
4. In which of the past 12 months, has this nose problem been accompanied by itchy-watery eyes? (Month names listed).
5. In the past 12 months, how much did this nose problem interfere with your daily activities? (Not at all, a little, a moderate amount, a lot)
6. Hayfever: Have you ever had hayfever? (Yes/No)

### The main independent variables

Information regarding exposure to traffic-related pollution was obtained through the following questions: how often do tucks pass near your home on weekdays? (Never, seldom, frequently through the day, almost all day).

### Confounders

A priori selection of the following confounding was done: sex (male/female), being born in Tembisa/Kempton Park the area (yes/no), type of house (brick, mud, corrugated iron, combination), vigorous physical activity (never/occasionally/1–2 times per week/, ≥ 3 times per week); How do you usually get to school? walk, taxi/bus, motorcar, combination of motorcar/taxi or train; hours watching television per day (<1 h/1 h but <3 h/ 3 h but <5 h /≥5 h) in 24 h; ETS exposure at home in the past 30 days (yes/no), ETS exposure at school in the past 30 days (yes/no), tobacco smoking by participant (yes/no), mother/father smoking tobacco (yes/no), any other person smoking at home other than participant (yes/no). Children were asked to select the most frequently used energy source at home; they had to select one type of energy source: for cooking at home (electricity, gas, paraffin, open fires) and for heating (electricity, gas, paraffin, open fires). Other variables, which were included in the questionnaire but not selected as confounders and were only reported in the descriptive analysis, were: period lived in the residential area (<6 months/ 6 to 12 months/ 1 to 2 years/ ≥3 years), the variables.

### Data management and statistical analysis

The data were entered into a database set up in EpiInfo V3.5.3. Stata Version 12 was applied for the data analysis. Prevalence rates for the health outcomes and proportion on risk factors under investigation were calculated by dividing the number of participants who responded affirmatively to a particular question by the number of questionnaires completed. Observations marked as “do not know”, “not stated” or “other responses” were set as missing. This resulted in each question having a slightly different sample size. Crude and adjusted odds ratios (OR) and 95 % confidence intervals (CI) were calculated with multilevel logistic regression analysis (MLRA) with random effect to estimate the likelihood of having rhinitis ever, current rhinitis, rhinoconjunctivitis and hayfever health outcomes given the presence of a potential risk factor. The multilevel data included sixteen schools nested within two districts (level 1). Confounding variables were added in a stepwise manner, starting with the most significant from the univariate analysis. Each time a new potential confounder was added to the model, if the effect estimate between the exposure of interest and respiratory outcome already in the models changed by more than 5 %, the additional variable was retained in the final multiple MLRA, otherwise the variable was removed and a different one was added [22]. This resulted in the final models having slightly different confounders. The most parsimonious multiple MLRA models were reported, i.e., those with variables having a p-value < 0.05 [22].

### Ethical considerations

The Ethics and Research Committee of the Faculty of Health Sciences, University of Pretoria approved the study (Ethics Number: S121/2011). The Gauteng Department of Education, Ekurhuleni North District, school principals and governing bodies were approached and gave approval and cooperation for the study. Parents of participants were sent a letter explaining the details and nature of the study and gave consent for the children to participate in the study. All information was kept confidential.

## Results

The study population consisted of 3764 children from 16 schools. A total of 3468 completed the questionnaires (92 % response rate). The study focused on children who were present at school during at the time of fieldwork and so 296 learners did not participate. The teachers gave assurance that most of the children were present. School attendance was high during the study therefore bias that may have been introduced by non-response rate was assumed to be relatively low. Forty-four questionnaires were excluded during the data capturing due to incomplete information. A total of 3424 questionnaires were finally included in the data analysis.

The frequencies and percentages for health outcomes and general characteristics of children are summarised in Table 1. The prevalence of rhinitis ever was 52.3 %, current rhinitis 40.1 % rhinoconjunctivitis 22.5 % and hayfever 37 %. Girls accounted for 52 % of the participants. The majority of the children lived in Tembisa Township (67.2 %) and more than three quarters had lived in the same area for more than 3 years (76.2 %). Fifty three percent of the children were born within the study areas. The majority of the children lived in formal housing structures (86.11 %). Forty one percent of the children engaged in vigorous physical activity once or twice per week, 29 % three or more times per week, the other 30 % never or occasionally. Forty two percent were exposed to tobacco smoke at home, whilst 34 % were exposed at school. A small percentage of pupils reported the use of gas (5.2 %) and paraffin (5.8 %) for cooking at home, while the majority used electricity (87.5 %). Trucks passing near residences almost all day were reported by 35.4 % of the children. Just over half of the children walked to school (50.5 %), while the other half used other modes of transport (cars, taxi, buses and train).

**Table 1:** Health outcomes and demographic characteristics of the study participants (*n* = 3424)

Variable	Total	Percentage
Rhinitis ever	1790	52.3
Current rhinitis	1372	40.1
Current rhinoconjunctivitis	769	22.5
Ever had hayfever	1285	37.5
Sex of child		
Female	1790	52.3
Male	1634	47.7
Type of Area		
Township	2301	67.2
Suburb	1117	32.6
Missing	6	0.18
Period lived in the area		
Less than 6 months	253	7.4
6 to 12 months	216	6.3
1 to 2 years	346	10.1
3 years and longer	2609	76.2
Born in the areas of Tembisa and Kempton Park		
Yes	1812	52.9
No	1609	47.0
Missing	3	0.1
Type of house		
Brick	2938	85.8
Mud	45	1.3
Corrugated iron	184	5.5
Combination	126	3.7
Missing	126	3.7
Vigorous physical activity per week		
Never or occasionally	984	28.7
Once or twice per week	1417	41.4
Three or more times a week	983	28.7
Missing	40	1.2
Hours watching television on average in a day		
Less than 1 h	532	15.5
Hour but less than 3 h	835	24.4
2 h but less than 5 h	827	24.4
More than 5 h	1213	35.4
Missing	17	0.50
ETS exposure at home in the past 30 days		
Yes	1452	42.4
No	1460	42.6
Missing	512	15.0
ETS exposure at school in the past 30 days		
Yes	1177	34.4
No	1452	42.4
Missing	755	23.2
Residential cooking fuel type most frequently used		
Electricity	2995	87.5
Gas	179	5.2
Paraffin	200	5.8
Open fires (wood, coal)	30	0.9
Missing	20	0.6
Residential heating fuel type most frequently used		
Electricity	2041	59.6
Gas	426	12.4
Paraffin	631	18.4
Open fires (wood, coal)	270	7.9
Missing	56	1.64
Mode of transport to school		
Walk	1728	50.5
Taxi\Bus	708	20.7
Motor car	683	20.0
Combination	201	5.9
Train	100	2.9
Missing	4	0.1
Frequency of trucks passing near homes on weekdays		
Never	563	16.4
Seldom	1033	30.2
Frequently through the day	580	16.9
Almost every day	1212	35.4
Missing	36	1.1

Table 2, summarise the MLRA results for the frequency of truck traffic and symptoms of rhinitis ever, current rhinitis, current rhinoconjunctivitis and hayfever along with crude and adjusted odd ratios (for further results see Appendix). Rhinitis ever; current rhinitis and current rhinoconjunctivitis were significantly associated with the frequency of trucks passing near residences almost all day on weekdays, (OR 1.46 95 % CI: 1.16 − 1.84), (OR 1.60 95 % CI: 1.24–2.02) and (OR 1.42 95 % CI: 1.09–1.84) respectively. No association was observed between truck traffic and hayfever in the multiple analyses.

**Table 2:** Frequency of truck traffic and symptoms of rhinitis ever, current rhinitis, current rhinoconjunctivitis and hayfever along with crude and adjusted odd ratios

Frequency of trucks passing near homes on weekdays	Total^e^	(%)	Crude OR (95 % CI)	*P*	Adjusted OR (95 % CI)	*P*
Rhinitis ever^a^						
Never	563	47.3	1		1	
Seldom	1033	53.1	1.26 (1.02–1.55)	0.027	1.16 (0.92–1.56)	0.197
Frequently through the day	580	49.3	1.09 (0.86–1.37)	0.451	1.03 (0.79–1.34)	0.791
Almost all day	1212	55.9	1.43 (1.16–1.75)	0.001	1.46 (1.16–1.84)	0.001
Current rhinitis^b^						
Never	563	34.6	1		1	
Seldom	1033	41.2	1.25 (1.01–1.55)	0.038	1.30 (0.99–1.62)	0.059
Frequently through the day	580	39.1	1.23 (0.97–1.57)	0.081	1.25 (0.95–1.64)	0.102
Almost all day	1212	42.5	1.46 (1.88–1.81)	0.000	1.60 (1.24–2.02)	0.000
Current rhinoconjunctivitis^c^						
Never	563	18.8	1		1	
Seldom	1033	23.0	1.23 (0.95–1.59)	0.113	1.17 (0.89–1.54)	0.234
Frequently through the day	580	19.0	1.01 (0.75–1.37)	0.904	0.96 (0.70–1.30)	0.799
Almost all day	1212	25.7	1.54 (1.20–1.97)	0.001	1.42 (1.09–1.84)	0.008
Hayfever^d^						
Never	563	32.7	1		1	
Seldom	1033	38.7	1.22 (0.98–1.51)	0.074	1.12 (0.87–1.43)	0.362
Frequently through the day	580	37.9	1.28 (1.00–1.63)	0.047	1.19 (0.90–1.57)	0.203
Almost all day	1212	38.2	1.34 (1.08–1.66)	0.007	1.23 (0.96–1.57)	0.087

^a, b^Model adjusted for the folowing v mode of transport to school, being born in the area, vigorous physical activity, ETS exposure at home in the past 30 days, residential fuel heating type most frequently used at home

^c^Model adjusted for mode of transport to school, being born in the area, vigorous physical activity, hours watching TV during normal week, residential heating fuel type most frequently used at home

^d^Model adjusted for vigorous physical activity, residential fuel heating type most frequently used at home, ETS exposure at home

^e^Totals for each risk factor are different due to difference in missing values

The following confounding variables were associated with the health outcomes: the likelihood of rhinitis ever, current rhinitis, current rhinoconjunctivitis and hayfever were less for males, (OR 1.22 95 % CI: 1.05–1.43), (OR 0.82 95 % CI: 0.70–0.96), (OR 0.68 95 % CI: 0.57–0.81) and (OR 0.56 95 % CI: 0.47–0.65). Rhinitis ever was associated with vigorous physical activity once or twice per week OR 1.27 95 % CI: 1.06–1.53), ETS exposure at home (OR 1.15 95 % CI: 0.99–1.34). Current rhinitis was associated with being born in the area OR 0.82 95 % CI: 0.70–0.96), vigorous physical activity once or twice per week OR 1.27 95 % CI: 1.05–1.53), the use of gas for heating OR 1.31 95 % CI: (1.04–1.65) and ETS at home (OR 1.16 95 % CI: 0.99–1.36). Rhinoconjunctivitis was associated with being born in the area (OR 1.22 95 % CI:(1.02–1.45), vigorous physical activity once or twice per week (OR 1.42 95 % CI: 1.16–1.76) and three or more times a week (OR 1.34 95 % CI: 1.06–1.68), watching TV per week day (OR 1.37 95 % CI: 1.06–1.78) and gas frequently used for heating at home (OR 1.45 95 % CI: 1.14–1.85). Hay fever, sex, vigorous physical activity once or twice per week (OR 1.38 95 % CI: 1.44–1.68) and three or more times per week (OR 1.52 95 % CI: 1.23–1.88), open fires used for heating (OR 1.51 95 % CI 1.13–2.02) and exposure to tobacco smoke at home (OR 1.20 95 % CI: 1.02–1.40). Rhinitis ever and current rhinitis were associated with using a combination of walking and taxi/bus as mode of transport to school; (OR 1.45 95 % CI: 1.04–2.04), (OR 1.81 95 % CI: 1.21–2.71). No association was observed between hayfever and rhinoconjunctivitis with mode of transport to school.

## Discussion

The study investigated the association between the frequency of truck traffic and allergic rhinitis symptoms, rhinoconjunctivitis and hayfever amongst 13 to 14 year old school children attending schools in areas of Tembisa and Kempton Park, located in EMM. The prevalence of rhinitis ever was 52.3 %, current rhinitis 40.1 %, rhinoconjunctivitis 22.5 % and hayfever 37 %. The prevalence of childhood allergic rhinitis shows wide variation throughout the world, ranging from 0.8 to 39.7 % [23]. The prevalence of rhinitis ever reported for this study is close to that reported for Turkish adolescents of 53.5 %, while current rhinitis was slightly higher at (38.3 %) [24], and similar to that in Bogotá, Colombia, which was 36.7 % [10]. The prevalence of current rhinitis in this study was high, in contrast to that (14.9 %) reported in Budapest [25] and to that reported in two previous studies conducted in Cape Town, South Africa, 30 % in 1999 and 38 % 2003 [18]. Twenty-two centres across the African continent participated in Phase III of the ISAAC study. There were considerable variations in the prevalence of allergic rhinoconjunctivitis within the participating centres (7.2–27.3 %). A number of centres showed high symptoms of allergic rhinoconjunctivitis (Cape Town, Reunion Island, Brazzaville, Eldoret, Urban Ivory Coast, Conakry, Casablanca, Wilays of Algiers and Sousse) [23].

Trucks passing near homes almost the whole day during weekdays increased the likelihood of rhinitis ever, current rhinitis and current rhinoconjunctivitis. Studies have previously reported on the association between traffic-related pollution and allergic rhinitis symptoms. A study conducted in Bochum, Germany reported a positive relationship between the prevalence of wheezing and allergic rhinitis and the indicators of traffic density [26]. A positive global relationship between childhood symptoms of rhinoconjunctivitis and self-reported truck traffic on the street of residence were reported from studies that were conducted in 110 ISAAC centres globally [27]. A cross-sectional study conducted amongst 32,143 Taiwanese school children found that allergic rhinitis was associated with urban levels of SO_2_, carbon monoxide (CO) and NO_X_ (traffic-related air pollution NOx and CO) [28]. A present report by the World Health Organization found evidence linking health effects to traffic-related pollution [29]. In recent years, South Africa, particularly Gauteng Province, has experienced an increase in the number of cars and trucks on the road. The total number of live vehicles (licenced) in Gauteng province, where the EMM is located was over 4.4 million, in April 2015 [30]. Vehicles, particularly trucks, are known to release polluting chemicals into the atmosphere, which may have respiratory effects on nearby residents. It is plausible that the observed association between health outcomes is linked to the increased level of pollution in the area.

Certain limitations should be taken into account in the interpretation of the results. Firstly, the study had a cross-sectional epidemiological design. The results of the study might be higher than the actual prevalence since the results are based on self-reported answers from the questionnaire. With cross-sectional studies, where a questionnaire is administered to collect data and participants are questioned on the presence of nasal symptoms, a significantly higher rate of symptoms is reported than for true allergic rhinitis [16]. However, cross-sectional studies are important indicators of health problems occurring in communities and serve as a baseline for further analytical and experimental investigation.

Secondly, we adjusted for confounding variable such as sex, being born in the area, physical activity, hours watching TV daily, mode of transport tot school; however there many other risk factors such as genetics, environmental factors and allergens which may play a role in the development and exacerbation of rhinitis. Thirdly, information on traffic density as an indicator of exposure to traffic related pollution was also taken from self-reports from children. The frequency of trucks passing near homes on weekdays may have been misclassified, as on weekdays children are at school. Furthermore, the traffic density may not accurately reflect the exposure the children experience inside and outside their homes. Children who are aware of the possible health effects of traffic-related air pollution and who have had symptoms may report exposure. Future studies should attempt to compare questionnaire responses with traffic data.

Fourthly, no quantitative air pollution exposures assessments were conducted during the study; we were not able to validate the participant’s responses by checking individual addresses or by measuring ambient pollution levels in or near their homes, as we did not have addresses. However, the findings are consistent with results of other studies, suggesting that traffic-related air pollution exacerbates existing conditions or increases the likelihood of the development of allergic rhinitis. Fifthly, although the study was done in an air pollution priority area, on the basis of multiple sources of air pollution; only proximity to truck traffic was investigated as an ambient (outdoor source) exposure variable. We did not include any other questions e.g., on distance of industries from residential areas. More research should be conducted in the area to investigate other outdoor air pollution sources. Studies should be conducted amongst children in the area to test for allergen triggering or exacerbating rhinitis symptoms.

Lastly the study was conducted from February to June 2012, in South Africa February to April falls is autumn, while May to July fall within winter, it is possible where data was collected in winter months children may have reported more symptoms than those surveyed during autumn months. Despite the limitations, there are also strengths which should be noted. The ISAAC questionnaire has been shown to be valid for this age group and has been used extensively in international studies relating to symptoms of rhinitis. Secondly, a larger sample size of more than 3000 children, as required by ISAAC centres, would increase the statistical power for the study.

## Conclusion

The study found an association between the frequency of trucks near residences and symptoms of rhinitis, rhinoconjunctivitis and hayfever 13 to 14 years old children, attending schools in Tembisa and Kempton Park located in EMM. This study will serve as a suitable baseline for monitoring future trends in the prevalence of allergic rhinitis amongst children in this particular area, which is known to have poor air quality.

## Appendix

**Table 3:** The prevalence of self-reported rhinitis ever along with crude and adjusted odd ratios

Variable	Total^a^	Rhinitis ever^a^ (%)	Crude OR (95 % CI)	*P*	Adjusted OR (95 % CI)^b^	*P*
Mode of transport to school						
Walk	1728	51.7	1		1	
Taxi/Bus	708	49.7	0.92 (0.77–1.10)	0.400	0.99 (0.81–1.21)	0.965
Motor car	683	53.9	1.08 (0.90–1.29)	0.358	1.15 (0.93–1.42)	0.186
Combination	201	60.8	1.42 (1.05–1.92)	0.019	1.45 (1.04–2.04)	0.028
Train	100	53.0	1.05 (0.70–1.58)	0.798	1.13 (0.71–1.81)	0.582
Frequency of trucks passing near homes on weekdays						
Never	563	47.3	1		1	
Seldom	1033	53.1	1.26 (1.02–1.55)	0.027	1.16 (0.92–1.56)	0.197
Frequently through the day	580	49.3	1.09 (0.86–1.37)	0.451	1.03 (0.79–1.34)	0.791
Almost all day	1212	55.9	1.43 (1.16–1.75)	0.001	1.46 (1.16–1.84)	0.001
Being born in Tembisa/Kempton park areas						
No	1609	49.3	1		1	
Yes	1812	54.9	1.28 (1.11–1.47)	0.000	1.22 (1.05–1.43)	0.008
Vigorous physical activity						
Never or occasionally	984	49.1	1		1	
Once or twice per week	1417	54.8	1.26 (1.07–1.48)	0.005	1.27 (1.06–1.53)	0.008
3 or more times per week	983	52.1	1.12 (0.94–1.34)	0.193	1.08 (0.88–1.31)	0.447
ETS exposure at home in the past 30 days						
No	1460	51.3	1		1	
Yes	1452	55.7	1.19 (1.03–1.38)	0.017	1.15 (0.99–1.34)	0.056
Residential heating fuel type most frequently used						
Electricity	2041	51.4	1		1	
Gas	426	56.3	1.23 (0.99–1.52)	0.055	1.23 (0.98–1.56)	0.070
Paraffin	631	52.9	1.07 (0.87–1.31)	0.487	1.04 (0.84–1.28)	0.694
Open fire	270	51.1	0.99 (0.76–1.28)	0.973	1.07 (0.80–1.14)	0.621

^a^Totals for each risk factor are different due to difference in missing values

^b^Model adjusted for all the variables in this table

**Table 4:** The prevalence of self-reported current rhinitis with crude and adjusted odd ratios

Variable	Total^a^	Current rhinitis (%)	Crude OR (95 % CI)	*P*	Adjusted OR (95 % CI)^b^	*P*
Mode of transport to school						
Walk	1728	37.1	1		1	
Taxi/Bus	708	40.1	1.11 (0.92–1.33)	0.253	1.15 (0.93–1.42)	0.194
Motor car	683	43.2	1.18 (0.92–1.51)	0.169	1.31 (0.92–1.87)	0.127
Combination	201	52.7	1.75 (1.27–2.43)	0.001	1.81 (1.21–2.71)	0.004
Train	100	44.0	1.27 (0.83–1.93)	0.259	1.40 (0.86–2.27)	0.172
Frequency of trucks passing near homes on weekdays						
Never	563	34.6	1		1	
Seldom	1033	41.2	1.25 (1.01–1.55)	0.038	1.30 (0.99–1.62)	0.059
Frequently through the day	580	39.1	1.23 (0.97–1.57)	0.081	1.25 (0.95–1.64)	0.102
Almost all day	1212	42.5	1.46 (1.88–1.81)	0.000	1.60 (1.24–2.02)	0.000
Sex						
Female	1790	43.0	1		1	
Male	1634	37.0	0.77 (0.67–0.89)	0.000	0.82 (0.70–0.96)	0.016
Born within the area						
No	1609	37.6			1	
Yes	1812	42.2	1.29 (1.12–1.49)	0.000	0.82 (0.70–0.96)	0.013
Vigorous physical activity						
Never or occasionally	984	37.2	1		1	
Once or twice per week	1417	42.2	1.22 (1.03–1.44)	0.017	1.27 (1.05–1.53)	0.012
3 or more times per week	983	40.2	1.10 (0.92–1.33)	0.263	1.08 (0.88–1.34)	0.424
Residential heating fuel type most frequently used						
Electricity	2041	40.3	1		1	
Gas	426	46.0	1.26 (1.02–1.56)	0.029	1.31 (1.04–1.65)	0.022
Paraffin	631	36.3	0.93 (0.76–1.13)	0.466	0.88 (0.70–1.11)	0.289
Open fire	270	39.2	1.01 (0.77–1.31)	0.934	1.09 (0.81–1.46)	0.553
Exposure to ETS at home						
No	1460	39.0	1		1	
Yes	1452	42.0	1.16 (1.00–1.34)	0.048	1.16 (0.99–1.36)	0.054

^a^Totals for each risk factor are different due to difference in missing values

^b^Model adjusted for all the variables in this table

**Table 5:** Prevalence of self-reported current rhinoconjunctivitis along with crude and adjusted odd ratios

Variable	Total^a^	Allergic rhino-conjunctivitis (%)	Crude OR (95 % CI)	*P*	Adjusted OR (95 % CI)^b^	*P*
Mode of transport to school						
Walk	1728	20.7	1		1	
Taxi/Bus	708	22.3	1.09 (0.88–1.35)	0.387	1.06 (0.84–1.33)	0.615
Motor car	683	24.6	1.25 (1.01–1.54)	0.033	1.19 (0.87–1.62)	0.260
Combination	201	26.4	1.53 (1.10–2.12)	0.011	1.44 (0.98–2.11)	0.054
Other	100	27.0	1.46 (0.92–2.30)	0.105	1.57 (0.96–2.57)	0.068
Frequency of trucks passing near homes on weekdays						
Never	563	18.8	1		1	
Seldom	1033	23.0	1.23 (0.95–1.59)	0.113	1.17 (0.89–1.54)	0.234
Frequently through the day	580	19.0	1.01 (0.75–1.37)	0.904	0.96 (0.70–1.30)	0.799
Almost all day	1212	25.7	1.54 (1.20–1.97)	0.001	1.42 (1.09–1.84)	0.008
Sex						
Female	1790	25.8	1		1	
Male	1634	18.9	0.67 (0.57–0.79)	0.000	0.68 (0.57–0.81)	0.000
Being born in Tembisa/Kempton park areas						
No	1609	20.7	1		1	
Yes	1812	24.0	1.27 (1.07–1.50)	0.004	1.22 (1.02–1.45)	0.024
Vigorous physical activity						
Never or occasionally	984	18.4	1		1	
Once or twice per week	1417	24.8	1.45 (1.19–1.78)	0.000	1.42 (1.16–1.76)	0.001
3 or more times per week	983	23.3	1.33 (1.06–1.65)	0.011	1.34 (1.06–1.68)	0.012
Hours watching TV during normal week						
Less than 1 h	532	19.6			1	
1 h but less than 3 h	835	19.8	0.97 (0.74–1.28)	0.873	0.93 (0.69–1.24)	0.622
3 h but less than 5 h	827	23.1	1.20 (0.91–1.57)	0.180	1.15 (0.87–1.53)	0.313
5 h or more	1213	25.1	1.37 (1.07–1.77)	0.012	1.37 (1.06–1.78)	0.016
Residential heating fuel type most frequently used						
Electricity	2041	21.5	1		1	
Gas	426	29.3	1.50 (1.18–1.89)	0.001	1.45 (1.14–1.85)	0.002
Paraffin	631	20.1	0.96 (0.76–1.22)	0.760	0.95 (0.75–1.22)	0.739
Open fires (wood, coal)	270	24.1	1.18 (0.87–1.59	0.276	1.18 (0.86–1.61)	0.291

^a^Totals for each risk factor are different due to difference in missing values

^b^Model adjusted for all the variables in this table

**Table 6:** Prevalence of self-reported hayfever along with crude and adjusted odd ratios, by risk or protective factors

Variable	Total^a^	Hayfever (%)	Crude OR (95 % CI)	p	Adjusted OR (95 % CI)^b^	p
Frequency of trucks passing near homes on weekdays						
Never	563	32.7	1		1	
Seldom	1033	38.7	1.22 (0.98–1.51)	0.074	1.12 (0.87–1.43)	0.362
Frequently through the day	580	37.9	1.28 (1.00–1.63)	0.047	1.19 (0.90–1.57)	0.203
Almost all day	1212	38.2	1.34 (1.08–1.66)	0.007	1.23 (0.96–1.57)	0.087
Sex of child						
Female	1790	43.2	1		1	
Male	1634	31.3	0.59 (0.51–0.68)	0.000	0.56 (0.47–0.65)	0.000
Vigorous physical activity per week						
Never or occasionally	984	30.7	1		1	
Once or twice per week	1417	40.2	1.51 (1.27–1.80)	0.000	1.38 (1.44–1.68)	0.001
Three or more times per week	983	40.5	1.48 (1.23–1.79)	0.000	1.52 (1.23–1.88)	0.000
Residential fuel heating type most frequently						
Electricity	2041	35.8	1			
Gas	426	43.0	1.30 (1.05–1.61)	0.015	1.20 (0.95–1.52)	0.121
Paraffin	631	35.8	1.15 (0.94–1.40)	0.160	1.11 (0.89–1.39)	0.330
Open fire	270	43.3	1.46 (1.12–1.89)	0.004	1.51 (1.13–2.02)	0.005
ETS exposure at home						
No	1460	36.1	1		1	
Yes	1452	40.1	1.23 (1.05–1.43)	0.007	1.20 (1.02–1.40)	0.022

^a^Totals for each risk factor are different due to difference in missing values

^b^Model adjusted for all the variables in this table

## Abbreviations

BC: Black carbon
CI: Confidence intervals
EMM: Ekurhuleni metropolitan municipality
ETS: Environmental tobacco smoke
OR: Odds ratio
ISAAC: International study of asthma and allergies in childhood
LRA: Logistic regression analysis
UFP: Ultra-fine particles
